# Draft Genome Sequence of *Frankia* sp. Strain CcI6, a Salt-Tolerant Nitrogen-Fixing Actinobacterium Isolated from the Root Nodule of *Casuarina cunninghamiana*

**DOI:** 10.1128/genomeA.01205-13

**Published:** 2014-01-16

**Authors:** Samira R. Mansour, Rediet Oshone, Sheldon G. Hurst, Krystalynne Morris, W. Kelley Thomas, Louis S. Tisa

**Affiliations:** Suez Canal University, Ismailia, Egypta; University of New Hampshire, Durham, New Hampshire, USAb

## Abstract

Members of the actinomycete genus *Frankia* form a nitrogen-fixing symbiosis with 8 different families of actinorhizal plants. We report a 5.57-Mbp draft genome sequence for *Frankia* sp. strain CcI6, a salt-tolerant nitrogen-fixing actinobacterium isolated from root nodules of *Casurina cunninghamiana* grown in Egyptian soils*.*

## GENOME ANNOUNCEMENT

Members of the genus *Frankia* are well known for their facultative lifestyles as free-living soil dwellers and as plant symbionts of wood angiosperms termed actinorhizal plants (1–3). Actinorhizal plants are ecologically important as pioneer community plants, are distributed worldwide under a broad range of ecological conditions, and have economic significance for land reclamation, reforestation, soil stabilization, landscaping, and fuel. Their symbiosis allows actinorhizal plants to colonize harsh environmental terrains under diverse ecological conditions. The genomes for representatives from four major lineages have been sequenced (4–9) and have provided vital baseline information for genomic approaches toward understanding these novel bacteria.

In arid and semiarid areas, the salinization of soils and groundwater is a serious problem (10). Egypt, which occupies the northeastern corner of Africa, has arid and semiarid climates and greatly suffers from high soil salinity. Among the actinorhizal plants, the genus *Casuarina* has been shown to have the ability to grow well under such conditions, and it is widely distributed in Egyptian soil. *Frankia* sp. strain CcI6 was isolated from root nodules of *Casuarina cunninghamiana* grown in Egypt and shown to reinfect *C. cunninghamiana, Casuarina glauca*, and *Casuarina equisetifolia* (11). This strain showed an increased level of NaCl tolerance (12). Based on 16S phylogenetic analysis, this strain is close to *Frankia* sp. strain CcI3, another narrow-host range symbiont, whose genome has been sequenced. *Frankia* sp. CcI6 has the potential to be used as a large-scale inoculum for *Casuarina* trees involved in land reclamation of Egyptian saline soils. *Frankia* sp. CcI6 was sequenced to increase our understanding of its salt tolerance mechanisms and to provide information about its potential ecological roles and interactions with actinorhizal plants.

The draft genome of *Frankia* sp. CcI6 was generated at the Hubbard Genome Center (University of New Hampshire, Durham, NH) using Illumina technology (13) techniques. A standard Illumina shotgun library was constructed and sequenced using the Illumina HiSeq 2000 platform, which generated 17,617,450 reads (260-bp insert size) totaling 1,665.7 Mbp. The Illumina sequence data were assembled using CLC Genomics Workbench (version 6.5.1) and AllPaths-LG (version r41043) (14). The final draft assembly contains 138 contigs, with an N_50_ of 103 kb. The total size of the genome is 5.6 Mbp, and the final assembly is based on 1,243.2 Mb of Illumina draft data, providing an average 222× coverage of the genome.

The high-quality draft genome of *Frankia* sp. CcI6 was resolved to 138 contigs consisting of 5,565,969 bp, with a G+C content of 67.6%. The assembled *Frankia* sp. CcI6 genome was annotated via the Integrated Microbial Genomes (IMG) platform developed by the Joint Genome Institute, Walnut Creek, CA (15), and resulted in 4,900 candidate protein-encoding genes, 46 tRNA genes, and 3 rRNA regions.

### Nucleotide sequence accession numbers.

This whole-genome shotgun project has been deposited at DDBJ/EMBL/GenBank under the accession no. AYTZ00000000. The version described in this paper is version AYTZ01000000.
